# A rare presentation of a spinal diffuse midline glioma in a child: a case report

**DOI:** 10.11604/pamj.2023.44.183.39885

**Published:** 2023-04-19

**Authors:** Zineb Isfaoun, Khadija Laasri, Manal Jidal, Nadia Cherradi, Adil Melhaoui, Latifa Chat, Siham El Haddad, Maria El Kababri, Mohamed El Khorassani, Amina Kili, Naoual El Ansari, Laila Hessissen

**Affiliations:** 1 Mohammed V University, Pediatric Hematology and Oncology Department, Children’s Hospital in Rabat, Rabat, Morocco,; 2 Mohammed V University, Radiology Department, Children’s Hospital in Rabat, Rabat, Morocco,; 3 Mohammed V University, Pathology Department, Children’s Hospital in Rabat, Rabat, Morocco,; 4 Mohammed V University, Neurosurgery Department, Children’s Hospital in Rabat, Rabat, Morocco

**Keywords:** Diffuse midline glioma, H3K27 altered, child, case report

## Abstract

Our patient had an extremely rare type of pediatric Diffuse Midline Glioma (DMG) with modified H3 K27 that occurred in the cervical spinal cord. Due to its location in the spinal cord, slow clinical presentation with torticollis for 7 months, and the non-specific radiological appearance of this tumour, it was initially considered to be a low-grade glioma. Based on imaging findings, the neurosurgery team performed a complete surgical resection, but the pathological features were consistent with a high-grade, diffuse midline glioma. Therefore, we are reporting a case of an altered high-grade DMG H3K27 glioma, which is difficult to diagnose due to its slow clinical symptoms which caused a delay in diagnosis, non-specific imaging, and with difficulty in accessing histopathological markers in low and middle income countries (LMIC).

## Introduction

Spinal cord tumors, especially high-grade tumors in pediatric patients, are rare. We report a case of diffuse midline glioma (DMG) with H3K27M alteration, which is a rare and highly aggressive tumor according to the 2021 World Health Organization (WHO) classification [1-3]. Our patient had an extremely rare type of pediatric DMG with modified H3 K27 that occurred in the spinal cord. Due to its medullary location, clinical presentation, and radiological appearance, the tumor was initially thought to be a low-grade glioma. Based on imaging results, surgical resection was performed by the healthcare team, but the pathological characteristics were different. We report a case of altered high-grade DMG H3K27 glioma with a difficult diagnosis due to slow clinical symptomatology with a neglected initial symptom which is torticollis, in addition to non-specific imaging, and difficult-to-access histopathological markers.

## Patient and observation

**Patient information:** a 6-year-old male patient with no notable personal or family pathological history presented with a neglected torticollis that had been present for seven months before admission. The evolution was marked by the aggravation of torticollis and the occurrence of left brachial monoparesis, which motivated consultation. The examination revealed altered left upper limb muscle strength, while muscle strength was preserved in the right upper limb and the lower limbs, and walking and standing were preserved without other neurological alterations. An MRI of the cervical spine revealed an intradural intramedullary tumor of the cervical spine. The patient underwent total tumor excision one month later. The patient was hospitalized in intensive care for three weeks, during which time he developed postoperative left hemiplegia and respiratory distress due to left diaphragmatic paralysis. This required a tracheotomy and progressive respiratory weaning, after which the patient was referred to the pediatric oncology department for further treatment.

**Clinical findings:** on examination, the patient was stable with regular spontaneous breathing, conscious, well oriented in time and space, in a wheelchair with left hemiplegia. Sensitivity was preserved, with no impairment of the cranial nerves, sensory or sphincter functions, and no *café-au-lait* spots or other abnormalities were found during skin examination.

**Figure 1:**
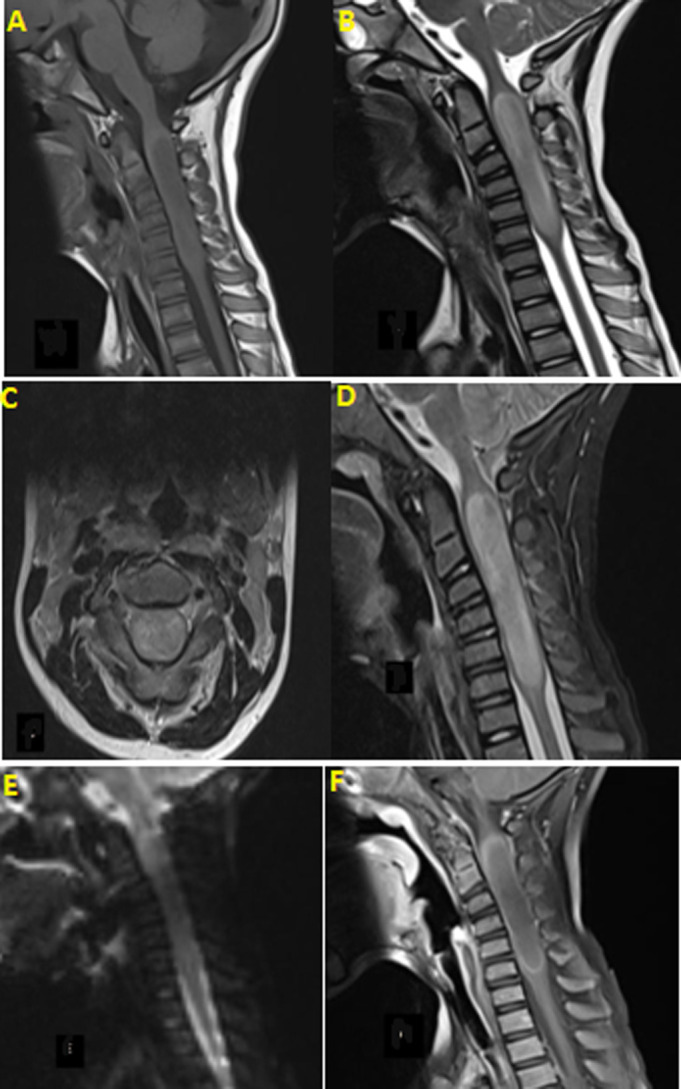
MRI images of the cervical spine; A) in sagittal T1; B) sagittal T2; C) axial T2; D) stir; E) diffusion; F) sagittal T1 after injection of gadolinium

**Figure 2:**
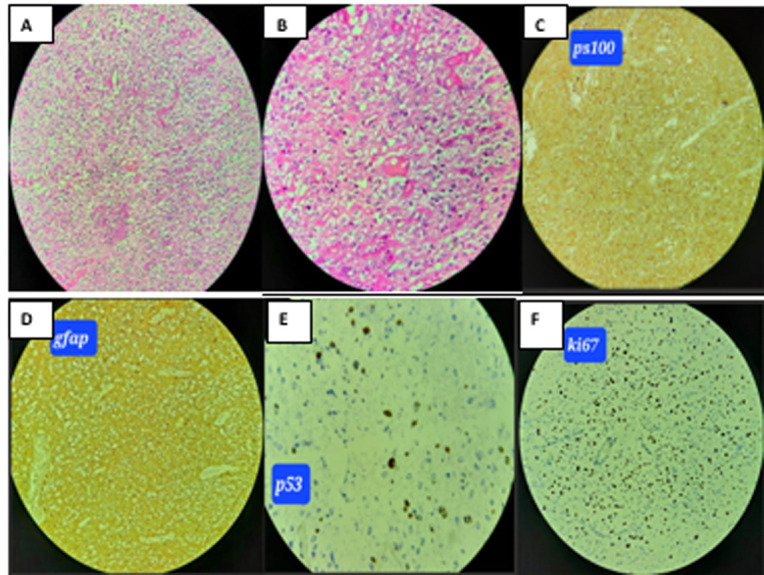
histomorphology and immunohistochemistry in H3 K27M-mt DMGs; A,B) diffuse infiltrating glioma; C) positivity of PS100; D) positivity of gfap; E) strong nuclear positivity of p53; F) Ki-67 index at 20%

**Timeline and diagnostic assessment:** at this stage, the diagnosis of low-grade glioma was evoked. The spinal MRI showed an expansive intramedullary intradural process, extended from C2 to C7, oblong, centered, well limited, in T1 iso-signal, heterogeneous hyper-signal in T2 and STIR, without diffusion restriction, enhanced peripherally after Gadolinium injection without central contrast, reducing the sub-arachnoid spaces, without bone lysis. Straightness of the cervical spine with beginning of inversion (Figure 1). The patient was operated, he benefited from a total excision of the tumor and the results of the anatomopathological study supported the diagnosis of diffuse midline glioma, with high levels of positivity for anti-GFAP, anti-PS100, and anti-ATRX. Positivity for anti-P53 was estimated at 10%, and positivity for anti-Ki67 was estimated at 20%. Anti-IDH1 was negative. An additional immunohistochemical study, which is difficult to access in LMIC countries, showed positivity for anti-H3K27M antibodies (Figure 2). Diffuse midline glioma H3K27 altered is classified as grade 4 according to the WHO 2021 classification, and has a poor prognosis.

**Therapeutic intervention:** the patient underwent complete tumor removal one month later. He was admitted to the intensive care unit for three weeks, during which he developed left hemiplegia and respiratory distress caused by left diaphragmatic paralysis after surgery. A tracheotomy and progressive weaning from mechanical ventilation were necessary. After recovery and confirmation of the diagnosis, the patient was referred for radiotherapy for the dose of 54Gy to be divided into 10 sessions.

**Follow-up and outcomes:** despite the poor prognosis associated with the presence of a high-grade H3K27M DMG subtype glioma located in the cervical spinal cord, the patient survived after undergoing total excision surgery, then he stayed in intensive care for hemodynamic and respiratory stability. When he woke up, the patient presented a left hemiplegia and then he was referred to radiotherapy for therapeutic follow-up.

**Informed consent:** the parents of the patient gave informed consent

## Discussion

We are reporting a case of a 6-year-old boy who suffered from neglected torticollis for 7 months. The patient only sought medical attention when monoparesis appeared, which ultimately led to a delayed diagnosis in this case. It is an uncommon presentation of a high-grade glioma with a slow progression and spinal localization. The MRI initially suggested a low-grade glioma, but the presence of anti-H3K27M antibodies in the immunohistochemical study confirmed the diagnosis of a diffuse midline glioma with a poor prognosis. According to the WHO 2021 classification, it is classified as grade 4. It is worth noting that LMIC countries face challenges in accessing markers for accurate diagnosis. In the literature, the rarer high-grade astrocytomas have a faster progression of symptoms. In contrast to our patient, who has mild symptoms. Paraspinal pain is the most common symptom, although radicular pain may be felt in some cases. Sensory disturbances may consist of dysesthesias, loss of proprioception and temperature sensation. However, these symptoms were not observed in our patient. In young children, the deterioration in gait, motor regression, kyphoscoliosis and torticollis are also important symptoms. In malignant tumors with painful symptoms, it follows with a rapid deterioration of motor function, culminating in disability within 3 to 5 months [4]. In contrast, our patient experienced a slow evolution with torticollis for seven months, followed by the development of monoparesis without clinical worsening.

The radiological diagnosis of DMG with H3K27M mutation of the spinal cord is difficult due to the lack of specific features on magnetic resonance imaging (MRI). The tumor appears hypo- to iso-signal on T1-weighted sequences and hyper-signal on T2-weighted sequences. In addition, the tumor margins are poorly defined, and slight peri-lesional edema may be notable. These radiologic features are consistent with high-grade glioma [5,6]. Some investigators have summarized that DMG H3K27M mutant arising in the cervical spinal cord typically exhibits homogeneous enhancement [7]. In our case, the limits were distinct, there was no peri-lesional edema, and the enhancement was peripheral. These features are different from those reported in the literature, but similar to the radiological features of spinal cord glioblastomas. Jo Sung Jung *et al*. [5] suggested that the presence of hemorrhagic areas is the only sign that can predict H3 K27M mutation. Differential diagnoses of DMG H3 K27M mutant of the spinal cord on imaging include ependymomas, astrocytomas, and hemangioblastomas [8].

In the WHO 2021 classification, the DMG with H3 K27 alteration, which was described in our clinical case, is characterized by a loss of nuclear expression of H3K27me3. This is the physiological form that is trimethylated on lysine (K) at position 27 of the H3 protein. This loss of expression is associated with either an H3 K27 mutation, an EGFR mutation, or an overexpression of EZHIP. Regardless of histopathological aspects, these gliomas are classified as grade 4 [1]. It is worth noting that in LMICs, there are challenges in accessing the necessary markers to accurately classify tumors. In our case, we faced difficulties in making a decision before obtaining the final marker. These markers play a significant role in the treatment of DMG. New therapeutic approaches that target DMG use a humanized anti-EGFR antibody, which has yielded results similar to those of more intensive chemotherapies, but with less of toxicity. It was used in combination with vinorelbine and radiotherapy, as well as in reirradiation by the Milan group. Other drugs, such as monoclonal antibodies, tyrosine kinase inhibitors and angiogenesis inhibitors, have also been described [9]. Our patient underwent surgery with complete excision, followed by radiation therapy.

## Conclusion

The new type of tumor, "diffuse midline glioma with H3K27 alteration," aims to establish a clearly defined and uniform diagnostic category that accurately describes the age, location, histology, and genetics of the tumor, with the ultimate goal of identifying this specific type of tumor. In our case, the discussion focused on the discrepancy between the slow clinical presentation with torticollis that resulted in a delayed diagnosis, the non-specific imaging for DMG, and the histological appearance, which indicates a high-grade tumor. However, characterizing tumors in LMICs is challenging due to the lack of specific markers.
